# *Gastrodin* Alleviates Oxidative Stress-Induced Apoptosis and Cellular Dysfunction in Human Umbilical Vein Endothelial Cells *via the Nuclear Factor-Erythroid 2-Related Factor 2*/Heme Oxygenase-1 Pathway and Accelerates Wound Healing *In Vivo*

**DOI:** 10.3389/fphar.2019.01273

**Published:** 2019-10-28

**Authors:** Jialiang Lin, Yifeng Shi, Jiansen Miao, Yuhao Wu, Hao Lin, Jianwei Wu, Weimin Zeng, Fangzhou Qi, Chen Liu, Xiangyang Wang, Haiming Jin

**Affiliations:** ^1^Department of Orthopaedics, The Second Affiliated Hospital and Yuying Children’s Hospital of Wenzhou Medical University, Wenzhou, China; ^2^Key Laboratory of Orthopaedics of Zhejiang Province, The Second Affiliated Hospital and Yuying Children’s Hospital of Wenzhou Medical University, Wenzhou, China; ^3^The Second School of Medicine, Wenzhou Medical University, Wenzhou, China

**Keywords:** gastrodin, wound healing, angiogenesis, apoptosis, *Nrf2*/HO-1

## Abstract

**Aims:** To explore the effect and mechanism of gastrodin (GAS) on human umbilical vein endothelial cells (HUVECs) apoptosis induced by oxidative stress and its function in wound healing.

**Main methods:** HUVECs were incubated with tert-butyl hydroperoxide (TBHP) to induce endothelial cell dysfunction and GAS was used as a protector. Cell viability was detected by Counting Kit-8 (CCK-8). HUVECs apoptosis was evaluated by TUNEL assay and western blotting for cleaved caspase3 (C-caspase3) and other apoptosis-related proteins. Transwell migration assay, tube formation assay, and cell-matrix adhesion assay were performed to evaluated cell function of HUVECs. Transfection with nuclear factor-erythroid 2-related factor 2 (*Nrf2*) small interfering ribonucleic acid and western blotting for *Nrf2*, HO-1, and apoptosis-related proteins were performed to prove that *Nrf2*/HO-1 pathway is involved in the protective effects of GAS. The skin wound model of rat was used to assess the protective effects of GAS *in vivo*.

**Key Findings:** The results show that treating HUVECs with GAS attenuated TBHP-induced apoptosis and cellular dysfunction, including cellular tube formation, migration, and adhesion. Mechanistically, we found that GAS protects HUVECs from TBHP-induced cellular apoptosis by activating the nuclear factor (erythroid-derived 2)-like 2 (*Nrf2*)/heme oxygenase 1 (HO-1) pathway. An *in vivo* study illustrated that the oral administration of GAS enhances vascularization in regenerated tissue and facilitates wound healing.

**Significance:** The findings of this study demonstrated that GAS may serve as a potential agent that accelerates wound healing.

## Introduction

The skin is a barrier to the outside world that protects the body from exogenous damage, and compared to other tissues, it is considered to be the most frequently injured tissue (Lee et al., 2006; Boer et al., 2016). Skin wounds caused by mechanical, chemical, electrical, and thermal injuries, as well as by chronic diseases, are a common health problem around the world; these wounds usually cause pain, infections, and even amputation in millions of patients, resulting in low quality of life and a high economic cost for society (Lee et al., 2006; Boer et al., 2016).

Cutaneous wound healing is a complex process that repairs and regenerates tissue structure and function; classically, this process can be divided into four highly integrated and overlapping phases: clot formation, inflammation, proliferation, and remodeling (Hosemann et al., 1991; Watelet et al., 2002; Mendonca and Coutinho-Netto, 2009; Velnar et al., 2009). This process is complex and involves the coordinated efforts of several cell types, including keratinocytes, fibroblasts, endothelial cells, macrophages, and platelets (Gurtner et al., 2008; Schultz et al., 2011; Wang et al., 2012). Angiogenesis, primarily carried out by endothelial cells, is reported to play a critical role in wound healing. During wound healing, the microvascular network comprising angiogenic capillaries can support cells at the wound site with nutrition and oxygen for wound healing (Tonnesen et al., 2000). However, while oxygen is utilized to produce energy *via* oxidative phosphorylation, reactive oxygen species (ROS) are produced and cause oxidative stress at the wound site, which can further cause endothelial injury (Rodriguez et al., 2008; Schafer and Werner, 2008; Kvietys and Granger, 2012; Scioli et al., 2014). Therefore, protecting endothelial cells from oxidative stress-induced damage may be a promising therapeutic target for accelerating cutaneous wound healing.

Heme oxygenase (HO)-1, a stress-response protein, serves as an inducible antioxidant enzyme and plays an important molecular role in host defence against oxidative stress (Choi and Alam, 1996; Araujo et al., 2012). In addition, endothelial cells are more vulnerable to damage in cases of heme oxygenase-1 (HO-1) deficiency, while HO-1 induction can reverse these effects (Kinderlerer et al., 2009; Taha et al., 2010). The regulation of HO-1 gene expression is linked to the transcription factor nuclear factor-erythroid 2-related factor 2 (*Nrf2*) (Vargas et al., 2005; Loboda et al., 2016). Activated *Nrf2* translocates to the nucleus and binds to antioxidant response elements, thus promoting the restoration of balance between oxidants and antioxidants after an oxidative insult. Consequently, we suggest that *Nrf2*/HO-1 pathway activation can be an effective therapeutic approach for protecting endothelial cells from oxidative stress-induced damage.

Gastrodin (GAS), a water-soluble phenolic glucoside with good oral bioavailability isolated from the traditional Chinese herbal *Gastrodia elata* (Yang et al., 2007), has been reported to possess anti-inflammatory (Wang et al., 2014), anti-nociceptive (Sun et al., 2012), and antioxidative effects (Jin et al., 2018). GAS protects against 1-methyl-4-phenyl pyridinium-induced oxidative stress by activating the *Nrf2*/HO-1 pathway in human dopaminergic cells (Jiang et al., 2014). *In vivo*, GAS was reported to ameliorate oxidative stress and proinflammatory responses in nonalcoholic fatty liver disease by regulating *Nrf2* expression (Qu et al., 2016). However, its effect on angiogenesis and wound healing remains unknown. Thus, we investigated the antioxidative effect of GAS on tert-butyl hydroperoxide (TBHP)-treated human umbilical vein endothelial cells (HUVECs) and explored the underlying mechanism. Furthermore, a full-thickness cutaneous wound rat model was used to evaluate whether GAS accelerates wound healing *in vivo*.

## Methods

### Reagents and Antibodies

GAS (purity >98%) was purchased from Nantong Feiyu Biological Technology Co., Ltd. (Nantong, China). GAS was dissolved in DMSO to create a 100 mM stock solution and then stored at −20°C. Tin protoporphyrin IX (SnPP), dimethyl sulfoxide (DMSO), TBHP, and carboxymethylcellulose (CMC) were purchased from Sigma-Aldrich (St. Louis, MO, USA). Primary antibodies against C-caspase3 (#9661), bax (#14796), Bcl-2 (#3498), caspase9 (#9508), cytochrome C (#11940), and β-actin (#3700) were obtained from Cell Signaling Technologies (Beverly, MA, USA). Primary antibodies against *Nrf2* (ab137550), HO-1 (ab189491), and lamin B (ab16048) were acquired from Abcam (Cambridge, UK), and 4’,6-diamidino-2- phenylindole (DAPI) was obtained from Beyotime (Shanghai, China). All cell culture reagents were purchased from Gibco (Grand Island, NY, USA).

### Cell Culture and Treatment Protocols

HUVECs were purchased from ATCC (Manassas, VA, USA) and grown in DMEM/F12 supplemented with 10% heat-inactivated FBS and 1% penicillin and streptomycin at 37°C in a humidified atmosphere of 5% CO2. To examine the effect of GAS on HUVECs viability, cells were treated with different concentrations of GAS for 24 h. To establish an in vitro oxidative stress and apoptosis HUVEC model, different concentrations of TBHP (100, 200, 500, and 1,000 μM) were added to the culture medium for 8 h to detect the cytotoxicity of TBHP. After determining the treatment concentration of TBHP, cells were pre-treated with different concentrations of GAS (5, 10, and 25 μM) for 2 h before TBHP addition to investigate the effects of GAS on cell apoptosis and dysfunction (**Figure S1**). To study the role of HO-1 in GAS-induced cell protection, endothelial progenitor cells were pre-treated with 20 μM SnPP, an HO-1 inhibitor, for 2 h prior to GAS treatment. All experiments were performed in triplicate.

### Cell Viability Assay

Cell viability was determined using a Cell Counting Kit-8 (Dojindo Co., Kumamoto, Japan) assay according to the manufacturer’s protocol. HUVECs were seeded in 96-well plates (3×10^3^ cell/well) and incubated with DMEM/F12 at 37°C for 24 h. Then, the cells were treated with various concentrations of GAS, TBHP, or GAS in combination with THBP as described above. After 24 h of treatment, the cells were washed with PBS. Then, 100 μl of non-FBS (DMEM/F12) containing 10 μl of Cell Counting Kit-8 solution was added to each well, and the plate was incubated for an additional 2 h. The absorbance of the wells at 450 nm was then measured by a microplate reader (Thermo Fisher, Waltham, MA, USA).

### Western Blotting

Western blotting was performed using routine protocols. Treated HUVECs were isolated using radioimmunoprecipitation assay buffer with 1 mM PMSF (phenylmethanesulfonyl fluoride), and the protein concentrations were measured using a BCA Protein Assay Kit (Beyotime). Nuclear and cytoplasmic proteins were isolated using the Nuclear and Cytoplasmic Extraction Kit from Pierce (Mountain View, CA, USA) according to the manufacturer’s protocol. Equal amounts of proteins were separated by sodium dodecyl sulfate-polyacrylamide gel electrophoresis (SDS-PAGE) and transferred onto polyvinylidene difluoride membranes (Bio-Rad, USA). The membranes were blocked with 5% skim milk at room temperature for 2 h and then incubated overnight at 4°C with one of the following primary antibodies: cleaved-caspase3 (C-caspase3), Bax, Bcl-2, cytochrome C, caspase9, β-actin, *Nrf2*, HO-1, or lamin B. Subsequently, the membranes were incubated with the corresponding secondary antibodies for 2 h at room temperature. The protein bands were then visualized using a ChemiDicTM XRS + Imaging System (Bio-Rad, USA). Eventually, the software of Image Lab (Bio-Rad, USA) was used to analyze the expression of each group of proteins.

### Terminal Deoxynucleotidyl Transferase Deoxyuridine Triphosphate Nick End Labeling Assay

To measure the apoptotic DNA, terminal deoxynucleotidyl transferase (TdT) deoxyuridine triphosphate nick end labeling (TUNEL) assays were performed with an *In situ* Cell Death Detection Kit (F. Hoffmann-La Roche Ltd., Basel, Switzerland) to detect double-stranded DNA cleavage. Briefly, the treated HUVECs were washed with PBS and fixed with paraformaldehyde (4%) for 20 min at room temperature. Then, the cells were incubated with 3% H_2_O_2_ and freshly prepared 0.1% Triton X-100 for 10 min. The cells were washed with PBS three times at every step. After washing, the cells were incubated with the TUNEL reaction mixture in a humidified chamber at 37°C for 1 h. The cell nuclei were stained with DAPI. Finally, the stained slices were examined by a fluorescence microscope (Olympus Inc., Tokyo, Japan). All pictures were taken at a magnification of 100 times.

### Cell Migration Assay

HUVEC migration was assessed using an 8-μm-pore polycarbonate membrane Boyden chamber insert in a transwell apparatus (Costar, Cambridge, MA, USA). HUVECs were treated with TBHP and GAS as described above. Then, the cells were detached using trypsin/EDTA, centrifuged, and re-suspended as single-cell solutions. In total, 4×10^4^ cells in 200 μl of non-FBS-containing DMEM/F12 were seeded on a transwell apparatus, and 700 μl of culture medium containing 1% FBS was added to the lower chamber. Following incubation of the cells for 18 h at 37°C in a 5% CO2 incubator, the membranes were washed with PBS three times and fixed with 4% paraformaldehyde. The Transwell apparatus was then stained using a hematoxylin solution, and the cells on the top surface of the insert were wiped away with cotton wool. Cells that migrated to the bottom surface of the insert were counted manually in three random microscopic fields (100×).

### Tube Formation Assay

HUVEC tube formation was carried out on Matrigel-coated chamber slides using an *in vitro* angiogenesis assay kit (Chemicon, Temecula, CA, USA). An ECMatrix gel solution was mixed with ECMatrix diluent buffer and placed in a μ-Slide plate at 37°C for 1 h to allow the matrix solution to solidify. HUVECs were pre-treated as described above and then harvested with trypsin/EDTA. Then, HUVECs (2×10^4^ cells) were seeded on the layer of previously polymerized Matrigel. The Matrigel culture was incubated at 37°C for 6 h. Tube formation was evaluated using a phase contrast microscope (40×) and quantified by counting the number of connected cells in randomly selected fields of each well.

### Cell-Matrix Adhesion Assay

HUVEC adhesion assays were performed in six-well plates. The slices in the plates were pre-coated with fibronectin (5 μg/ml) at 37°C for 1 h. The cells were treated with TBHP and GAS before these assays were performed. Equal numbers of harvested cells (10^5^ cells/well) were seeded in the coated plates and allowed to adhere at 37°C for 30 min. Non-adherent cells were washed off with PBS and subsequently fixed with 4% paraformaldehyde. Adherent cells were detected by staining with DAPI. Three independent representative fields were assessed in each well, and the average numbers of adherent cells were determined.

### Small Interfering Ribonucleic Acid Transfection

Double-stranded siRNA for human *Nrf2* gene silencing was designed and chemically synthesized (RiboBio, Guangzhou, China). The *Nrf2* siRNA sequence was as follows: sense strand 5’-CGTCATTGATGATGAGGCT-3.’ Cells were transfected with 50 nM siRNA and Lipofectamine 2000 Reagent (Thermo Fisher, UT, USA) for 36 h according to the manufacturer’s instructions. Then, the cells were treated with GAS and TBHP as described previously, and the cells were harvested for western blot analysis after these procedures.

### Animal Experiments

Eight-week-old male SD rats were purchased from the Animal Center of the Chinese Academy of Sciences, Shanghai, China, and divided randomly into two groups (control group and GAS group, n = 18). All mice were anaesthetized intraperitoneally with 2% (w/v) pentobarbital (40 mg/kg) and monitored by an assistant during the surgery. After the dorsal area was shaved, two round full-thickness dermal wounds of 12.56 cm^2^ (2.0 cm diameter) were made on both sides of the dorsal trunk using fine scissors. The GAS group received GAS (50 mg/kg) dissolved in CMC intragastrically once per day from the day of surgery until the mice were sacrificed. Mice in the control group were administered an equivalent volume of CMC. At the end of 7, 14, and 21 days after wounding, the mice from each group (n = 6) were sacrificed under pentobarbital sodium anaesthesia, and the wounds and surrounding tissues were harvested for histological evaluation.

### Histological Analysis

Skin tissues were fixed in 4% paraformaldehyde overnight. Then, gross specimens were paraffin-embedded and sectioned to 5-mm thickness with a microtome. Slides for each specimen were stained with hematoxylin and eosin (H&E). Images were captured with an optical microscope. Then, the length of the wound closure and the number of capillaries in the wound bed were analyzed.

### Immunohistochemical Examination

The day 7 tissue sections embedded in paraffin were deparaffinized with heat, immersed in xylene, rehydrated, and washed with distilled water. Endogenous peroxidase activity was blocked by placing the sections in 3% hydrogen peroxide for 10 min. Then, 10% normal goat serum was used to block the nonspecific binding sites for 30 min at room temperature. The sections were then incubated with a primary antibody (anti-alpha smooth muscle actin (αSMA), 1:200) overnight at 4°C, followed by incubation with a Texas red-conjugated anti-IgG secondary antibody. The nuclei with counterstained with DAPI. All fluorescent images were taken using a fluorescence microscope (Olympus Inc., Tokyo, Japan).

### Statistical Analysis

All experiments were performed at least three times. The data obtained are expressed as the mean ± standard error of the mean (SEM). Statistical analyses were performed using GraphPad Prism version 5.0 software (GraphPad Software, San Diego, CA, USA). Inter-group comparisons were performed using a one-way ANOVA followed by the Tukey test. Probability values of P < 0.05 were considered statistically significant.

## Results

### Gastrodin Treatment Decreases Tert-Butyl Hydroperoxide-Induced Apoptosis in Human Umbilical Vein Endothelial Cells

**Figure 1:**
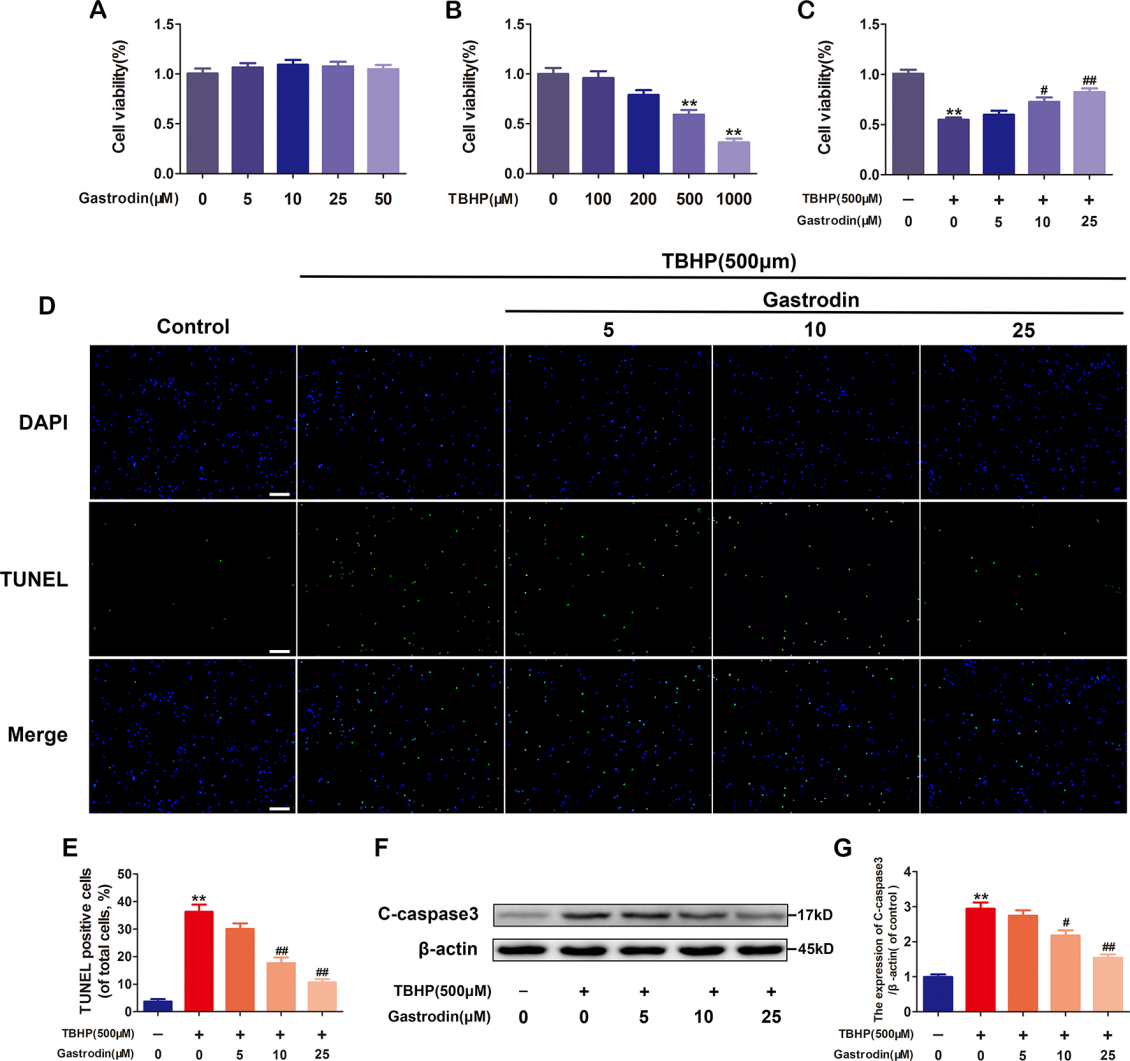
Gastrodin (GAS) treatment decreases tert-butyl hydroperoxide (TBHP)-induced apoptosis in human umbilical vein endothelial cells (HUVECs). **(A)** Cell Counting Kit-8 (CCK-8) results for HUVECs treated with different concentrations of GAS for 24 h. **(B)** CCK-8 results for HUVECs treated with different concentrations of TBHP for 8 h. **(C)** CCK-8 results for GAS-treated HUVECs stimulated with TBHP. **(D)** Representative images demonstrating terminal deoxynucleotidyl transferase deoxyuridine triphosphate nick end labeling-positive nuclei (green color). Scale bar, 100 μm. **(E)** Percentages of TUNEL-positive cells relative to total cells. **(F**, **G)** Representative western image and quantification data for C-caspase3 protein expression in the different groups. The data are presented as the mean ± SEM, **P< 0.01 relative to the control group. ^#^P < 0.05, ^##^P < 0.01 relative to the TBHP-stimulated group. n = 3.

Cell cytotoxicity was calculated as a percentage of the control group. As shown in **Figure 1**, no significant cytotoxicity in HUVECs was observed for the concentrations of GAS that were used. Under TBHP treatment, HUVEC viability decreased in a dose-dependent manner, and at a concentration of 500 μM, a 48% decrease in cell viability was observed; GAS pretreatment had a protective effect against TBHP-induced cell death (**Figures 1B, C**). To further determine the anti-apoptosis effect of GAS on TBHP-treated HUVECs, TUNEL and western blotting assays were performed. The results showed that the rate of TUNEL-positive cells and the level of C-caspase3, an apoptosis-related protein, were increased by TBHP stimulation but reversed by GAS pretreatment in a dose-dependent manner (**Figures 1D–G**).

### Gastrodin Alleviates Mitochondrial Functional Damage in Tert-Butyl Hydroperoxide-Treated Human Umbilical Vein Endothelial Cells

**Figure 2:**
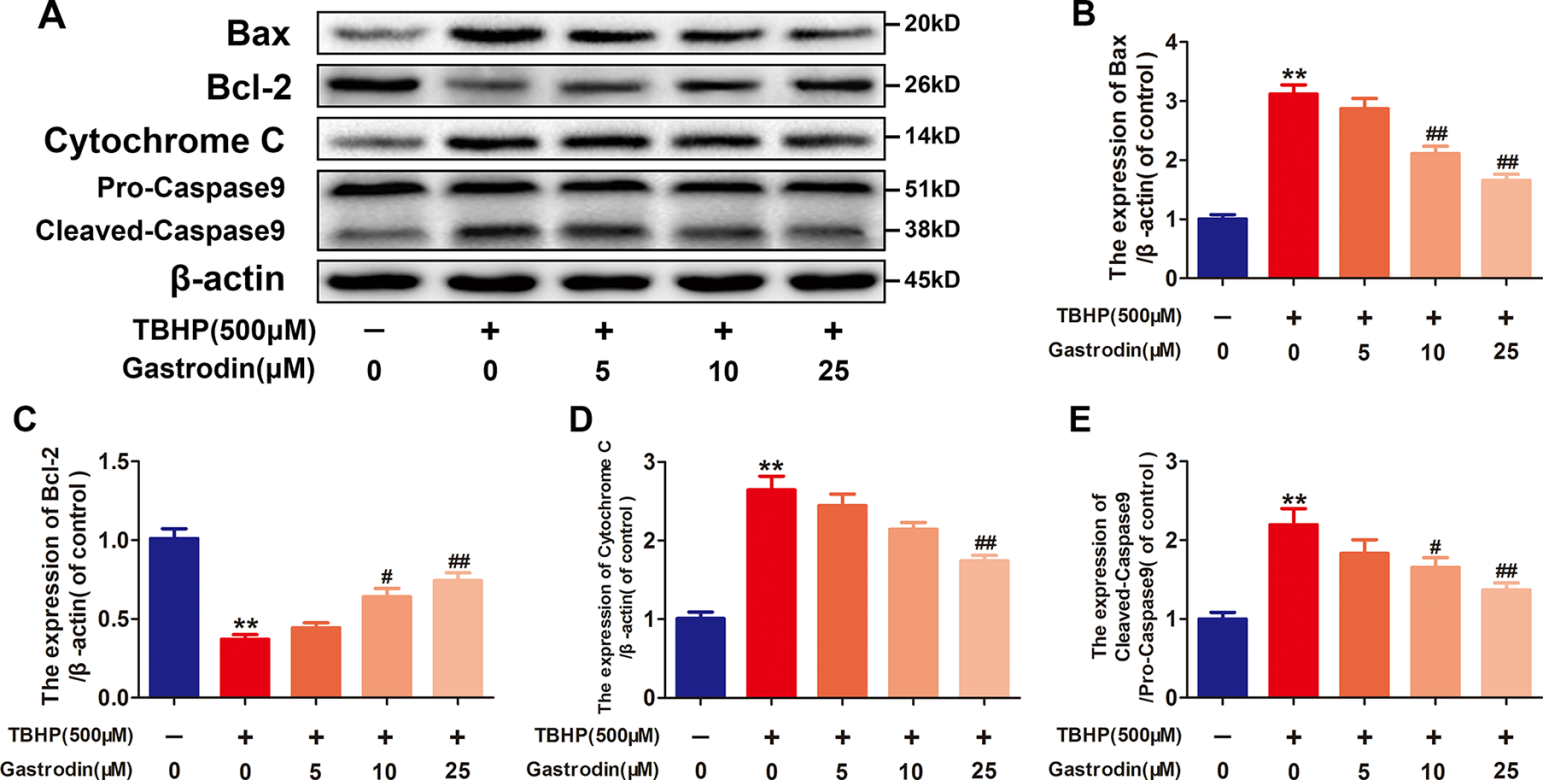
Gastrodin (GAS) alleviates mitochondrial functional damage in tert-butyl hydroperoxide (TBHP)-treated human umbilical vein endothelial cells (HUVECs). **(A**–**E)** Representative western blot images and quantification data for Bax, Bcl-2, cytochrome C, and caspase9 protein expression in HUVECs treated with GAS and TBHP. The data are presented as the mean ± SEM, **P < 0.01 relative to the control group. ^#^P < 0.05, ^##^P < 0.01 relative to the TBHP-stimulated group. n = 3.

As shown in **Figure 2**, the western blotting analysis results revealed that TBHP-induced expression changes in mitochondria dysfunction markers, including Bax, Bcl-2, caspase9, and cytochrome C, in HUVECs were antagonized by GAS pretreatment in a dose-dependent manner. Furthermore, GAS significantly decreased the level of intracellular ROS in TBHP-treated HUVECs (**Figure S2**).

### Gastrodin Improves Cell Function in Tert-Butyl Hydroperoxide-Treated Human Umbilical Vein Endothelial Cells

**Figure 3:**
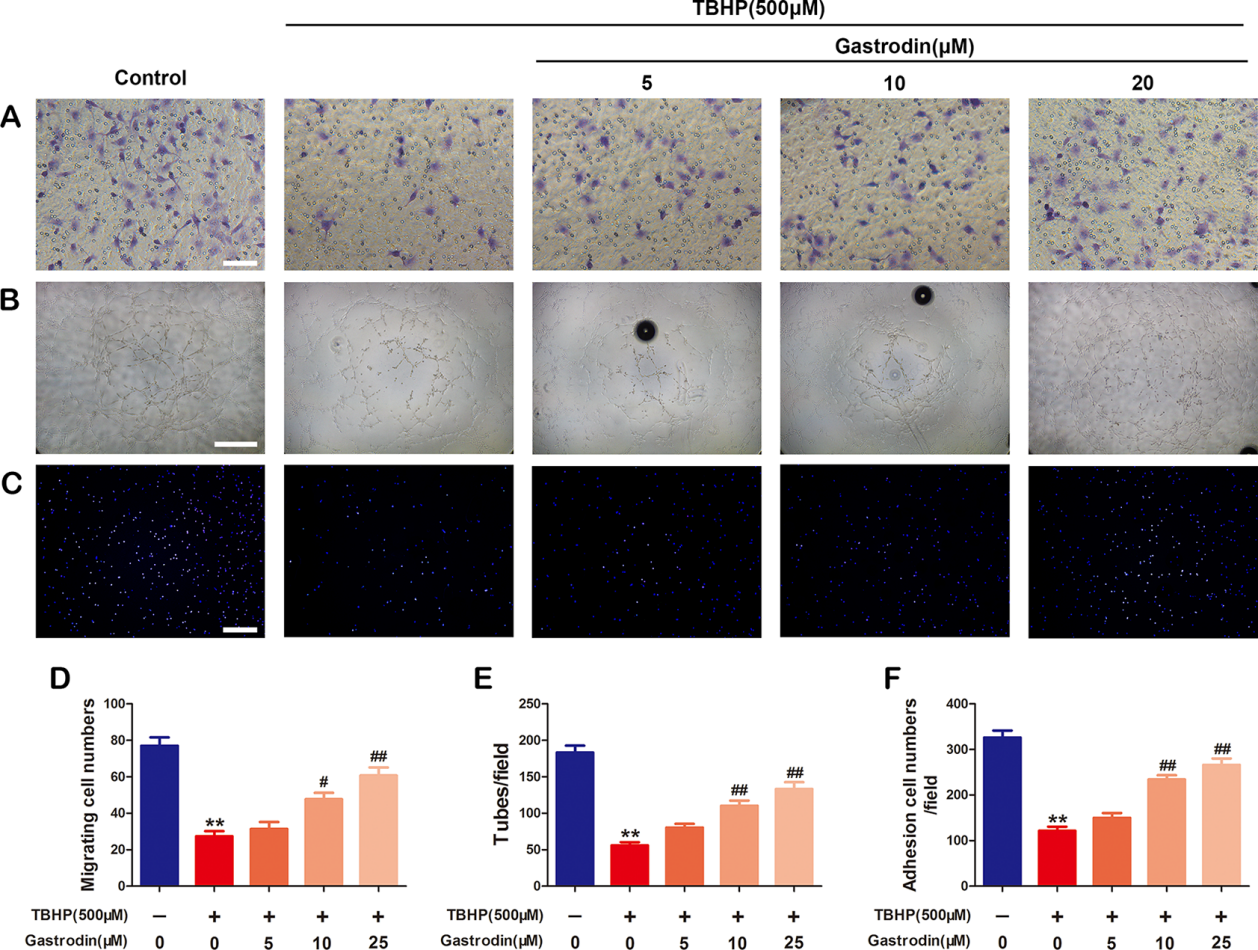
Gastrodin (GAS) improves cell function in tert-butyl hydroperoxide (TBHP)-treated human umbilical vein endothelial cells (HUVECs). **(A**, **D)** Transwell migration assay results demonstrating the effect of GAS on HUVEC migration. Scale bar, 100 μm. **(B**, **E)** Tube formation assay results demonstrating the effect of GAS on HUVEC neovascularization. Scale bar, 200 μm. **(C**, **F)** Cell-matrix adhesion assay results demonstrating the effect of GAS on HUVEC adhesive function. Scale bar, 200 μm. The data are presented as the mean ± SEM, **P < 0.01 relative to the control group. ^#^P < 0.05, ^##^P < 0.01 relative to the TBHP-stimulated group. n = 3.

The effects of GAS treatment on cell migration, tube formation, and adhesion in TBHP-treated HUVECs were investigated. First, a transwell migration assay was used to investigate the effect of GAS on HUVEC migration. **Figure 3** shows representative images of hematoxylin-stained migratory cells in the basolateral membranes. Treatment with TBHP significantly reduced the number of migratory HUVECs. Compared to TBHP treatment alone, GAS effectively protected TBHP-treated HUVECs, and significantly increased the migratory cell number (**Figure 3**). Second, to study the effects of GAS on HUVEC neovascularization, tube formation assays were performed, and the numbers of capillary-like structures in the groups were counted. The results showed that TBHP significantly suppressed HUVEC neovascularization, while GAS pretreatment protected against the effects of TBHP in a dose-dependent manner (**Figures 3B, E**). Finally, a fibronectin adhesion assay was used to evaluate the effect of GAS on HUVEC adhesive function. The statistical results indicated that the number of adherent cells was decreased by TBHP stimulation, but these effects were reversed by GAS pretreatment (**Figures 3C, F**).

### Anti-Apoptotic Effects of Gastrodin Are Related to Heme Oxygenase-1 Expression in Tert-Butyl Hydroperoxide-Treated Human Umbilical Vein Endothelial Cells

**Figure 4:**
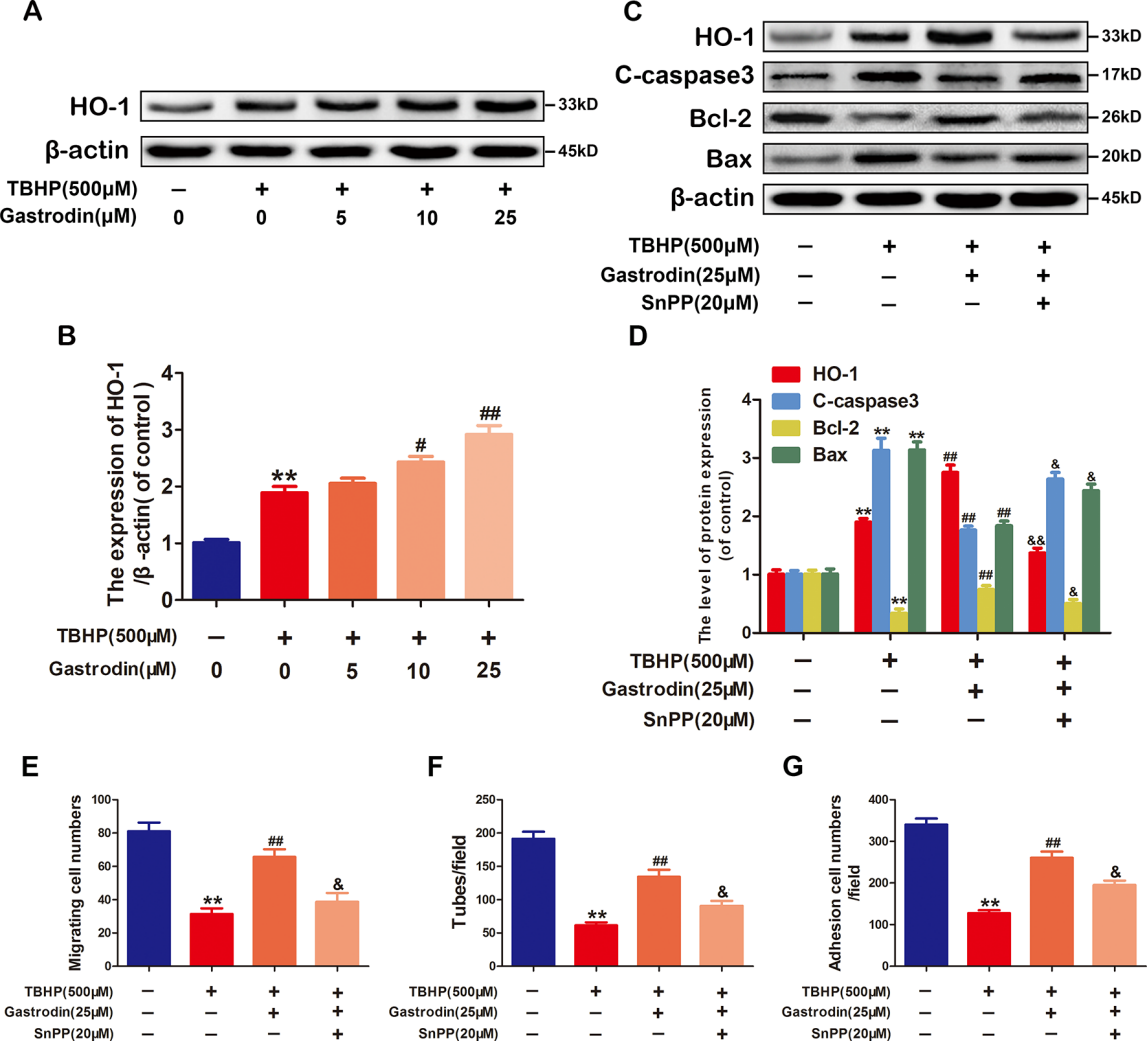
The anti-apoptotic effect of gastrodin (GAS) is related to heme oxygenase-1 (HO-1) expression in tert-butyl hydroperoxide (TBHP)-treated human umbilical vein endothelial cells. **(A**, **B)** Representative western blot image and quantification data for HO-1 protein expression in each group treated as described above. **(C**, **D)** Representative western blot image and quantification data for HO-1, C-caspase3, Bax, and Bcl-2 for each group treated with TBHP, GAS, and protoporphyrin IX (SnPP). **(E**–**G)** The results of the cell migration, tube formation, and adhesion assays for each group treated with TBHP, GAS, and SnPP. The data are presented as the mean ± SEM, **P < 0.01 relative to the control group. ^#^P < 0.05, ^##^P < 0.01 relative to the TBHP-stimulated group. ^&^P <0.05, ^&&^P < 0.01 relative to the TBHP plus GAS co-treated group. n = 3.

To investigate whether GAS exerts anti-apoptotic properties in HUVECs by inducing HO-1 expression, we first analyzed HO-1 protein expression after treatment with GAS at various concentrations. Although GAS increased HO-1 expression in a dose-dependent manner compared with TBHP treatment only, it failed to achieve statistical significance at 5 μM (**Figures 4A, B**). Next, the cells were pre-treated with the HO-1 inhibitor SnPP to examine whether HO-1 was involved in the melatonin-induced protective effect in HUVECs. GAS-induced cytoprotection against TBHP exposure was weakened by the inhibition of HO-1 activity using SnPP (**Figures 4C, D**). Third, the protective effects of GAS on cell migration, tube formation, and adhesion were reversed by the HO-1 inhibitor SnPP (**Figures 4**). Taken together, these data suggest that the upregulation of HO-1 expression is required for the anti-apoptotic effects of GAS on TBHP-treated HUVECs.

### The Nuclear Translocation of *Nrf2* Increases Heme Oxygenase-1 Expression

**Figure 5:**
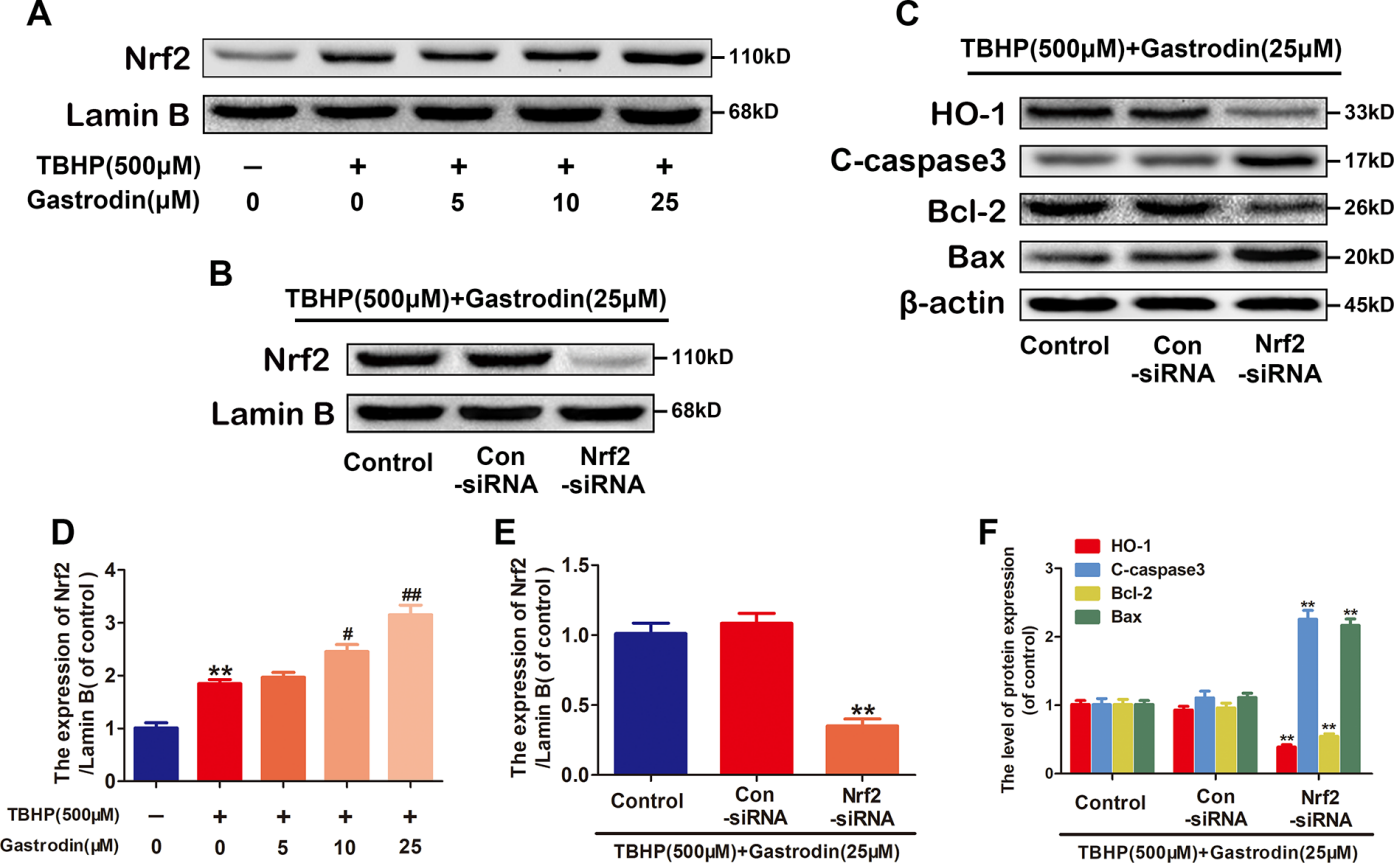
The nuclear translocation of nuclear factor-erythroid 2-related factor 2 (*Nrf2*) increases heme oxygenase 1 (HO-1) expression. **(A**, **D)** Representative western blot image and quantification data for nuclear *Nrf2* protein expression for each group treated as described above. **(B**–**F)** After transfection with *Nrf2* small interfering ribonucleic acid, the protein expression levels of *Nrf2* in the nucleus and HO-1, C-caspase3, Bax, and Bcl-2 in the cytoplasm of *Hypericum perforatum* ethyl acetate (HUVECs) co-treated with tert-butyl hydroperoxide (TBHP) and gastrodin (GAS) were analyzed by western blotting. The data are presented as the mean ± SEM, **P < 0.01 relative to the control group. ^#^P < 0.05, ^##^P < 0.01 relative to the TBHP-stimulated group. n = 3.

Activated *Nrf2* translocates to the nucleus and binds to an antioxidant response element (ARE), which promotes the expression of ARE-related antioxidant genes. To investigate whether GAS upregulates HO-1 *via Nrf2*, we first analyzed the nuclear *Nrf2* protein levels in treated HUVECs. Compared with TBHP treatment alone, treatment with GAS increased the nuclear levels of *Nrf2* (**Figures 5A, D**). Furthermore, RNA interference (RNAi) against *Nrf2* was performed to assess its involvement in the protective effects of GAS on TBHP-induced cell apoptosis. The western blotting results showed that *Nrf2* siRNA markedly suppressed nuclear *Nrf2* expression and decreased cytoplasmic HO-1 expression in TBHP and GAS co-treated HUVECs. In addition, the GAS-induced changes in apoptosis-related proteins were reversed by *Nrf2* siRNA as expected (**Figures 5B–F**). TUNEL assay also confirmed that *Nrf2* siRNA abolished the protective effects of GAS (**Figure S3**). Taken together, these data indicate that *Nrf2* upregulates HO-1 expression and mediates the protective effects of GAS on HUVECs under TBHP stimulation.

### Gastrodin Accelerates Cutaneous Wound Healing in Rats

**Figure 6:**
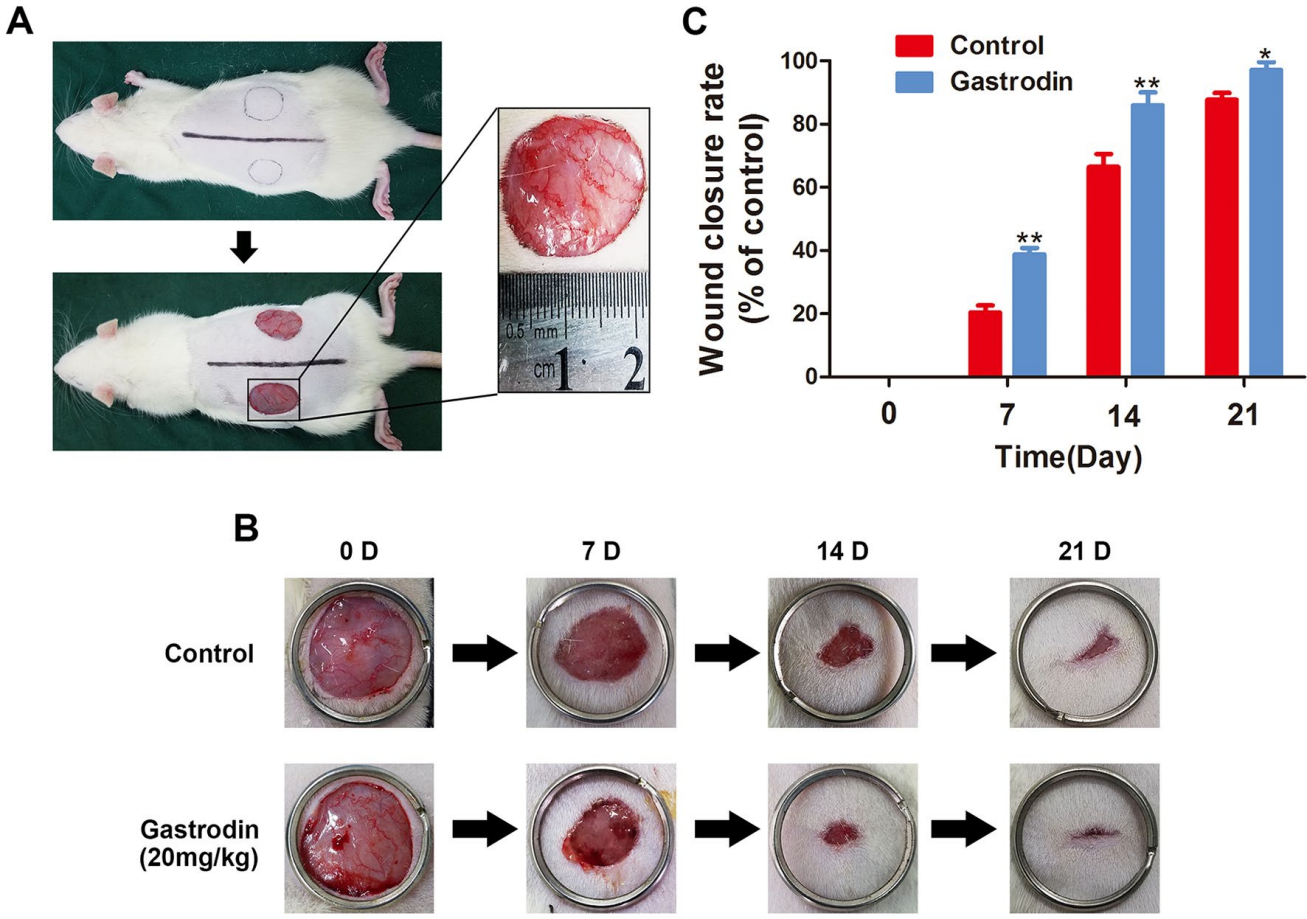
Gastrodin (GAS) accelerates wound closure. **(A)** Two round, full-thickness dermal wounds were made on both sides of the rat dorsal trunk. **(B)** Representative images of wound closure are shown for the control and GAS groups at days 0, 7, 14, and 21. **(C)** Wound closure rates for the two groups at different times. The data are presented as the mean ± SEM, *P < 0.05, **P < 0.01 relative to the control group on the same indicated day. n = 6.

**Figure 7:**
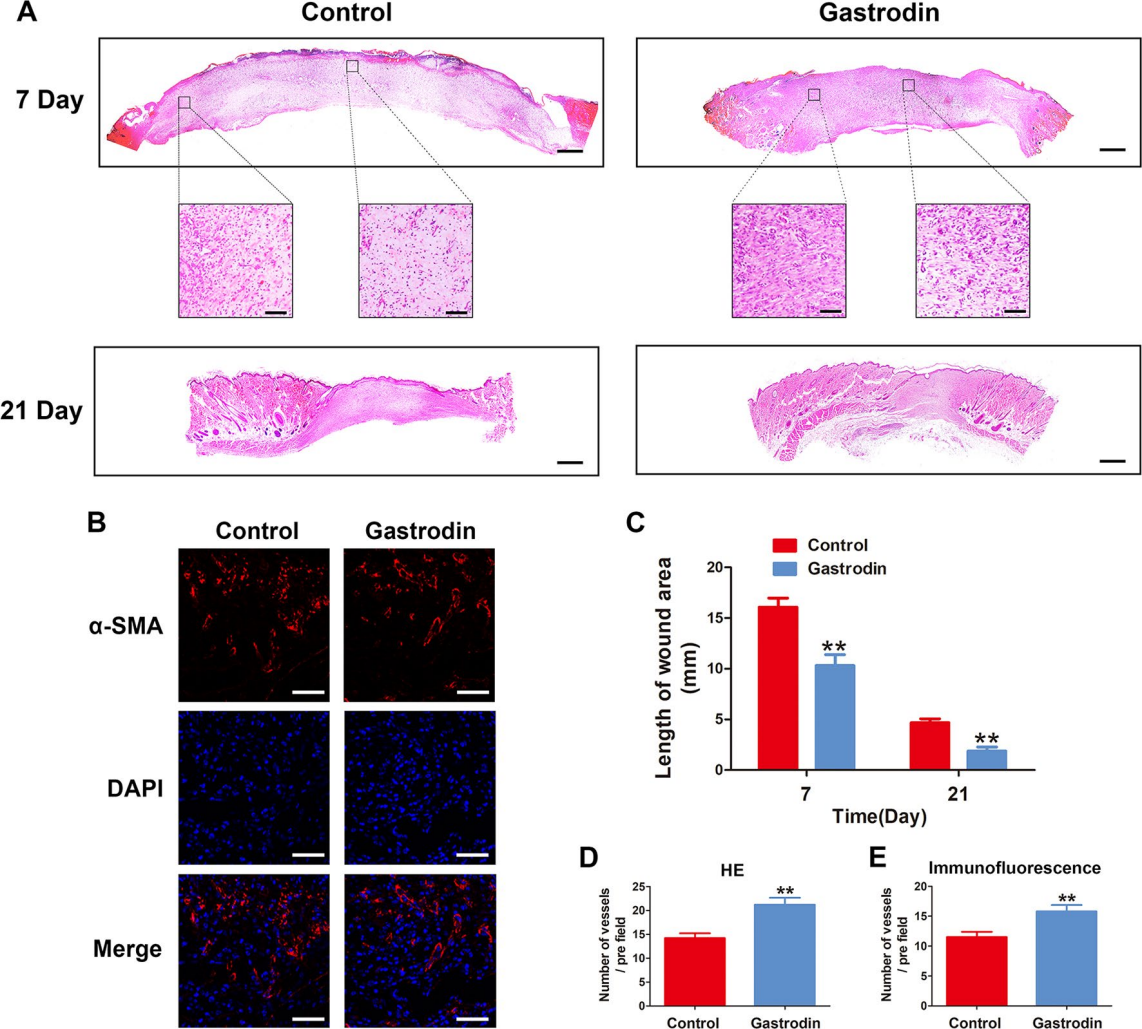
Gastrodin (GAS) promotes the wound healing process. **(A)** Representative images of hematoxylin and eosin (H&E)-stained sections of wounds at 7 and 21 days post-operation. Scale bar, 500 μm. Scale bar in the enlarged images, 100 μm. **(B)** Blood vessels in the wound bed were observed by alpha smooth muscle actin (αSMA) staining on day 7. Scale bar, 100 μm. **(C)** Wound lengths for the two groups at days 7 and 21. **(D**, **E)** Quantification data for the number vessels per field on day 7 in the αSMA-stained and H&E-stained enlarged images. The data are presented as the mean ± SEM, **P < 0.01 relative to the control group on the same indicated day. n = 6.

The surgical procedure is shown in **Figure 6**. The wound closure was smaller in the GAS-treated group than in the control group on days 7, 14, and 21, and significant differences were found between these groups based on the analysis results (**Figures 6B, C**). Moreover, H&E staining also showed that the GAS-treated group had a smaller wound area than the control group (**Figures 7A, C**). The enlarged images of the H&E staining and αSMA staining also show that the wound bed had strikingly more capillaries on the 7th day after GAS treatment (**Figures 7A, B**). Furthermore, GAS significantly decreased ROS level *in vivo* (**Figure S4**). Collectively, these results indicate that GAS could accelerate cutaneous wound healing in rats.

## Discussion

In this study, we provided evidence that GAS treatment activated the *Nrf2*/HO-1 pathway in HUVECs, which subsequently alleviated oxidative stress-induced apoptosis and improved the cellular function of HUVECs. In addition, the *in vivo* study suggested that GAS may promote wound healing in a rat full-thickness cutaneous wound model.

There is an intricate cascade of events involved in skin repair, and the wound healing process is regulated by a large variety of different growth factors, cytokines, and hormones (Barrientos et al., 2008; Schultz et al., 2011; Behm et al., 2012). In addition, ROS and ROS signalling play vital roles not only in disinfection during the inflammatory phase but also in other phases, including migration, proliferation, and angiogenesis (Steiling et al., 1999; Schafer and Werner, 2008). Low levels of ROS are essential for stimulating effective wound healing. However, the presence of excess levels of ROS causes oxidative stress, leading to the apoptosis of cells, including endothelial cells, in the surrounding tissues (Steiling et al., 1999; Lum and Roebuck, 2001; auf dem Keller et al., 2006). GAS, a natural water-soluble compound extracted from the root of Gastrodin elata, has been shown to have potent antioxidative activity (Jiang et al., 2014; Li and Zhang, 2015; Jin et al., 2018). Therefore, we investigated the protective effect of GAS in alleviating TBHP-induced cell injury.

In this study, we found that GAS (< 50 μM) did not effect on cell viability significantly. Then, we used TBHP to induce oxidative stress in HUVECs. Similar to the results of other studies, we confirmed that TBHP significantly decreased viability and induced apoptosis in HUVECs, whereas pretreatment with GAS reversed these effects. It implies that GAS may affect cell viability under TBHP stimulation. Furthermore, the results also showed that GAS could attenuate the upregulation of bax, cytochrome C, and cleaved caspase9, as well as decrease Bcl-2 expression. These results suggest that the mitochondrial pathway may be involved in the anti-apoptotic effects of GAS. In addition, the protective effect of GAS treatment on tube formation, migration, and adhesion in TBHP-treated HUVECs was also investigated. We demonstrated that pretreatment with GAS could improve the impaired cell function induced by TBHP in HUVECs.

Inducing HO-1 is widely recognized as an important therapeutic target in the pharmacological intervention of vascular disease (Durante, 2010). HO-1 is highly expressed in tissues such as the heart and blood vessels, and it plays an important role in protecting against vasculopathy and has cytoprotective effects on the circulation (Dulak et al., 2004; Hull et al., 2013; Issan et al., 2014). HO-1-deficient mice, however, are more susceptible to severe vascular damage (True et al., 2007; Taha et al., 2010). *Nrf2* is a nuclear factor that has been reported to control the expression of detoxifying enzymes, and the activation of *Nrf2* could upregulate HO-1 expression (Loboda et al., 2016). Under physiological conditions, Kelch-like ECH-associated protein-1 (Keap1) constitutively targets *Nrf2* for ubiquitin-dependent proteasomal degradation. After triggering exogenous stimuli, Keap1 is inactivated, and the ubiquitination degradation of *Nrf2* be weaken, which leads to the accumulation of *Nrf2* and allows it to enter the nucleus and bind to antioxidant responsive elements (Motohashi and Yamamoto, 2004; Lu et al., 2016; Yamamoto et al., 2018). This process results in the increased production of antioxidants and phase II detoxification enzymes, such as HO-1. Oxidative stress could induce the activation of the *Nrf2*/HO-1 pathway, which plays a role in maintaining homeostasis in the face of oxidative injury. However, although HO-1 expression and *Nrf2* accumulation in the nucleus were increased in TBHP-stimulated HUVECs, cell apoptosis and dysfunction still occurred. This phenomenon may be due partly to the insufficient activation of the *Nrf2*/HO-1 pathway to prevent toxicity under TBHP stimulation. It has been reported that GAS can ameliorate oxidative stress-induced damage through the *Nrf2*/HO-1 pathway in particular cell types (Zhang et al., 2018). Indeed, the results of our study demonstrated that GAS significantly increased *Nrf2* accumulation in the nucleus and HO-1 expression in TBHP-treated HUVECs. However, treatment with the HO-1 inhibitor SnPP attenuated the protective effects of GAS in TBHP-treated HUVECs. *Nrf2* siRNA transfection also suppressed the expression of *Nrf2*, which subsequently abolished the upregulation of HO-1 expression and increased the expression of apoptosis-related proteins in HUVECs. These results suggest that the protective effects of GAS in TBHP-treated HUVECs were mediated mainly by the activation of the *Nrf2*/HO-1 pathway.

To verify the therapeutic effects of GAS in vivo, a rat full-thickness cutaneous wound model was established in our study, and the wound healing process was evaluated after 0, 7, 14, and 21 days of successful modelling. The results of our study showed that GAS sped up the healing process. The GAS-treated group had a higher wound closure rate than the control group. By day 21, the wounds in the GAS-treated group were completely closed, while some of the wounds in the control group remained unhealed. Quantitatively, the vessel numbers were significantly higher in the GAS treatment groups than in the control group. However, it is worth noting that angiogenesis is closely related to tumorigenesis. Whether GAS will increase cell viability and promote tumorigenesis *in vivo* treatment remains to be further studied. Taken together, these findings of this study suggest that GAS may serve as a potential agent that accelerates wound healing.

## Conclusions

In conclusion, based on the *in vivo* and *in vitro* results of our study, GAS is suggested to be a promising intervention that can accelerate wound healing. The protective effects of GAS on endothelial fibrosis were accomplished by alleviating oxidative stress-induced apoptosis and improving cellular function in endothelial cells. The activation of the *Nrf2*/HO-1 pathway may play a key role in wound healing treatment using GAS.

## Data Availability Statement

The datasets generated for this study are available on request to the corresponding author.

## Ethics Statement

The animal study was reviewed and approved by Animal Care and Use Committee of Wenzhou Medical University.

## Author Contributions

XW, HJ, and JL contributed to the conception of the study. JL, YS, and JM were involved in analyzing the data and drafting the manuscript, and all authors were involved in revising it critically. YW and HL performed the cell experiment. JW and WZ performed the animal experiment, FQ contributed to the collection of data. HJ and CL provided important intellectual and technical contribution. XW was responsible for the acquisition of funding.

## Funding

This work was supported by the National Natural Science Foundation of China (81371988), the Major Scientific and Technological Project of Medical and Health of Zhejiang Province (WKJ-ZJ-1527), the Zhejiang Provincial Public Welfare Science and Technology Project (2017C33100), and the Zhejiang Provincial Natural Science Foundation of China (LY17H060008).

## Conflict of Interest

The authors declare that the research was conducted in the absence of any commercial or financial relationships that could be construed as a potential conflict of interest.
