# Supplementary material for: Gastrodin Alleviates Oxidative Stress-Induced Apoptosis and Cellular Dysfunction in Human Umbilical Vein Endothelial Cells via the Nuclear Factor-Erythroid 2-Related Factor 2/Heme Oxygenase-1 Pathway and Accelerates Wound Healing In Vivo
Source: Front Pharmacol. 2019 Oct 28;10:1273. doi: 10.3389/fphar.2019.01273 (PMC6843024; doi:10.3389/fphar.2019.01273)
Supplement: Supplementary file 1 [file DataSheet_1.docx]

**Supplementary materials**

**Fig. S1.** **A schematic diagram for the cell viability experiment (Fig. 1C).**


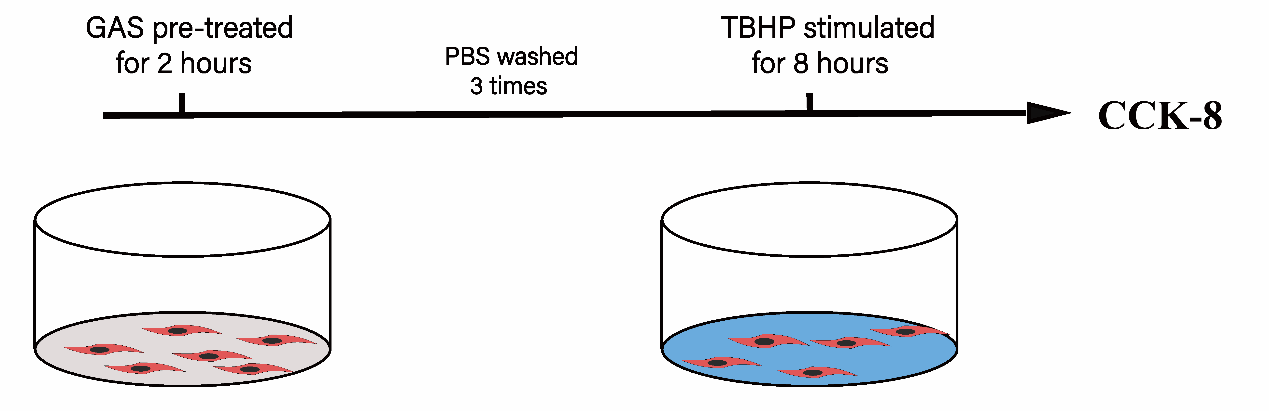


**Fig. S2**


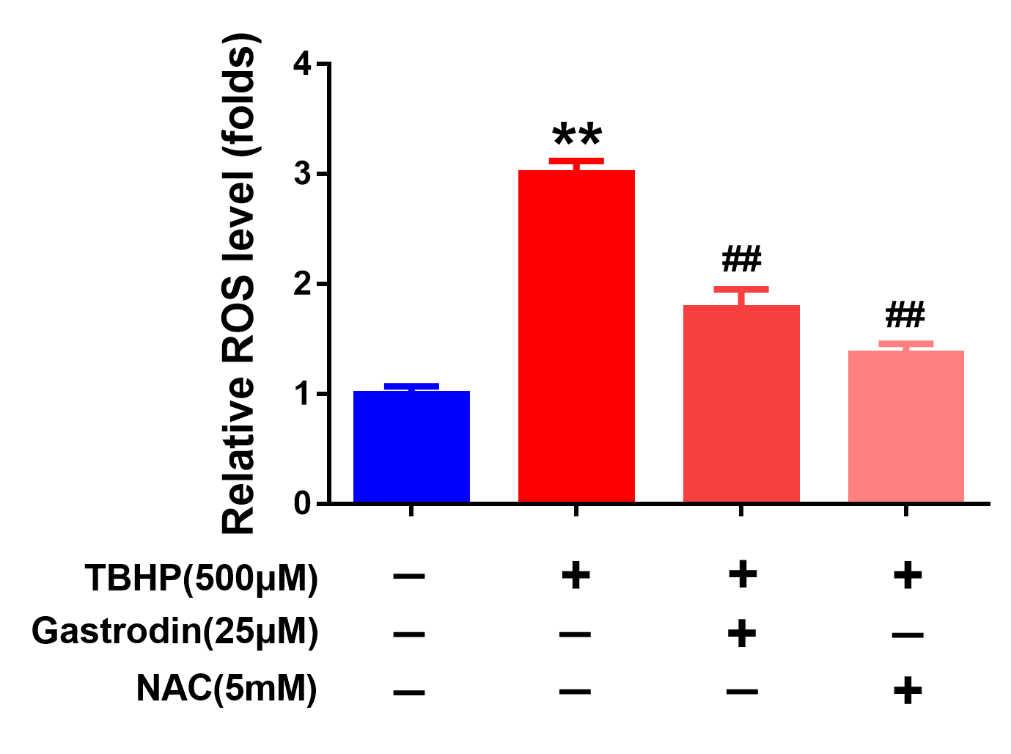


**Fig. S2: GAS decreases the level of intracellular ROS in HUVECs under oxidative stress.** The level of intracellular ROS generation was detected in HUVECs treated above. The data are presented as the mean ± SEM, **P<0.01 relative to the control group. ##P<0.01 relative to the TBHP-stimulated group. n=3.

**Fig. S3**


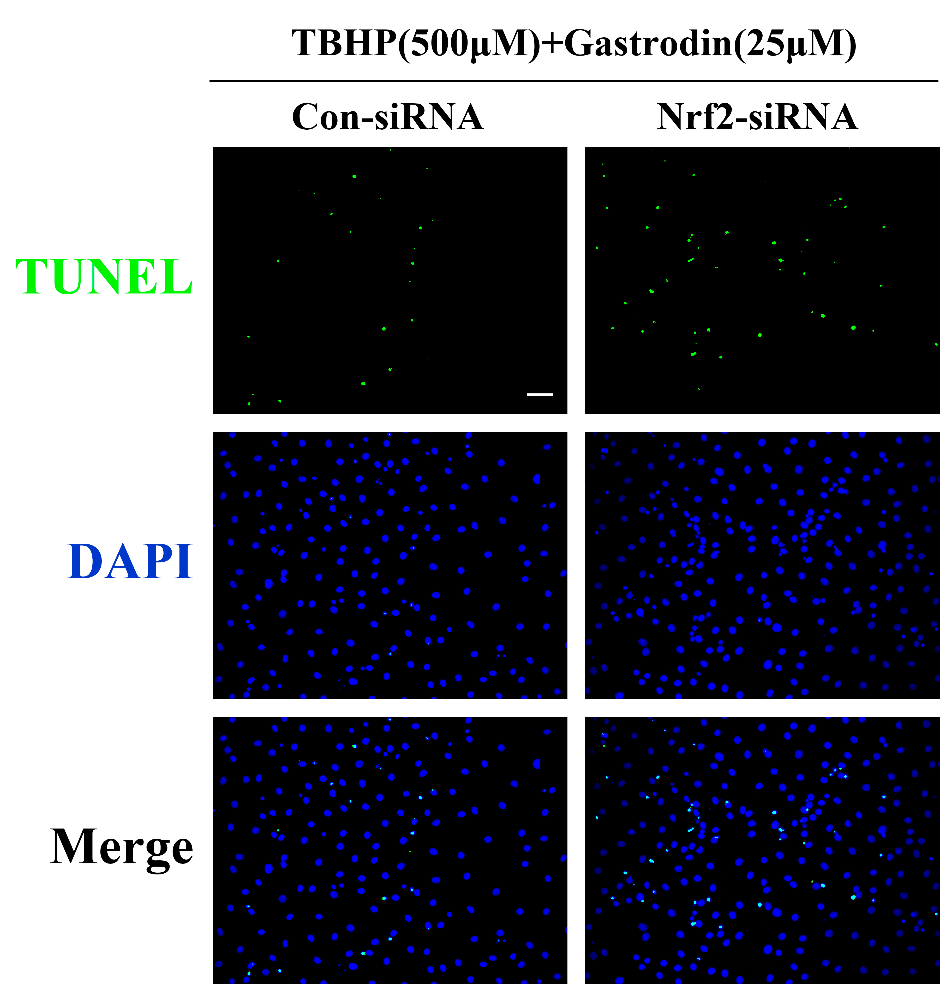

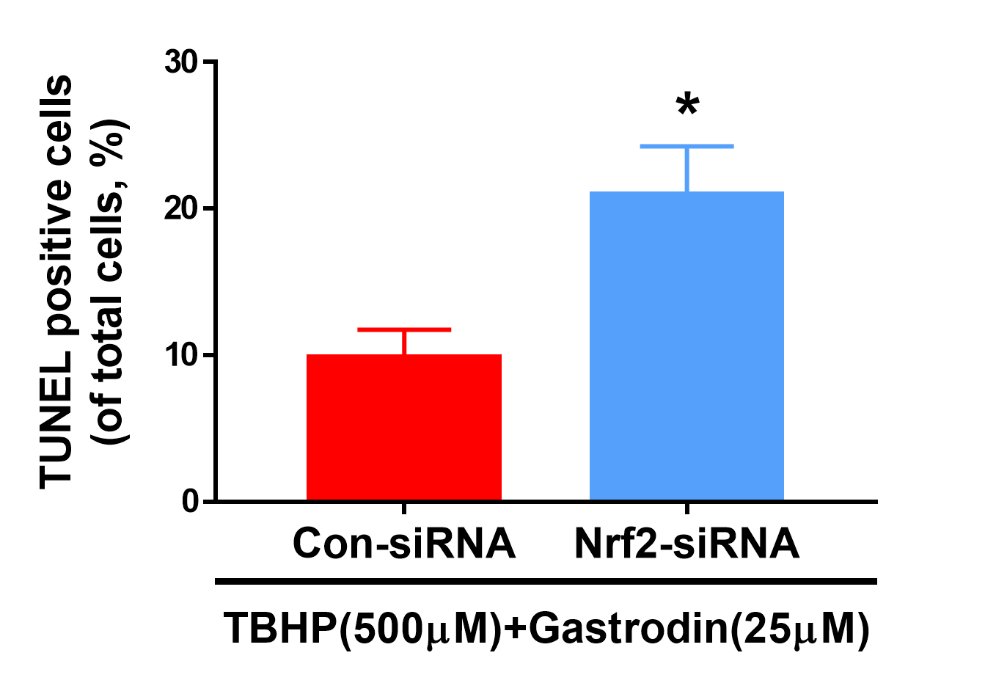


**Fig. S3: Knocking down Nrf2** **alleviates the protective effects of GAS.** Tunel assay was performed to detect the apoptosis of HUVECs treated above. The data are presented as the mean ± SEM, *P<0.05 relative to the Con-siRNA group. n=3.

**Fig. S4**


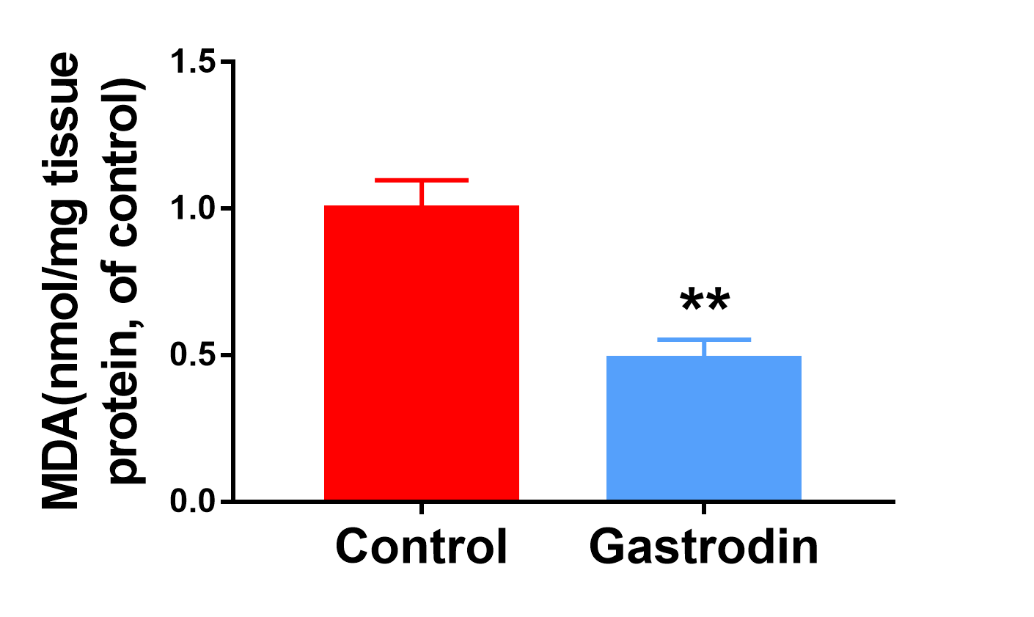


**Fig. S4: GAS decreases the ROS level *in vivo*.** The MDA assay was performed to detect the level of oxidative stress marker MDA in wound tissues at 7 days post-operation. The data are presented as the mean ± SEM, **P<0.01 relative to the control group on the same indicated day. n=6.
